# Incremental Yield of Including Determine-TB LAM Assay in Diagnostic Algorithms for Hospitalized and Ambulatory HIV-Positive Patients in Kenya

**DOI:** 10.1371/journal.pone.0170976

**Published:** 2017-01-26

**Authors:** Helena Huerga, Gabriella Ferlazzo, Paolo Bevilacqua, Beatrice Kirubi, Elisa Ardizzoni, Stephen Wanjala, Joseph Sitienei, Maryline Bonnet

**Affiliations:** 1 Epicentre, Paris, France; 2 Médecins Sans Frontières, Paris, France; 3 Institute of Tropical Medicine, Antwerp, Belgium; 4 Médecins Sans Frontières, Nairobi, Kenya; 5 Ministry of Health, Nairobi, Kenya; 6 IRD UMI 233 TransVIHMI - UM - INSERM U1175, Montpellier, France; McGill University, CANADA

## Abstract

**Background:**

Determine-TB LAM assay is a urine point-of-care test useful for TB diagnosis in HIV-positive patients. We assessed the incremental diagnostic yield of adding LAM to algorithms based on clinical signs, sputum smear-microscopy, chest X-ray and Xpert MTB/RIF in HIV-positive patients with symptoms of pulmonary TB (PTB).

**Methods:**

Prospective observational cohort of ambulatory (either severely ill or CD4<200cells/μl or with Body Mass Index<17Kg/m^2^) and hospitalized symptomatic HIV-positive adults in Kenya. Incremental diagnostic yield of adding LAM was the difference in the proportion of confirmed TB patients (positive Xpert or MTB culture) diagnosed by the algorithm with LAM compared to the algorithm without LAM. The multivariable mortality model was adjusted for age, sex, clinical severity, BMI, CD4, ART initiation, LAM result and TB confirmation.

**Results:**

Among 474 patients included, 44.1% were severely ill, 69.6% had CD4<200cells/μl, 59.9% had initiated ART, 23.2% could not produce sputum. LAM, smear-microscopy, Xpert and culture in sputum were positive in 39.0% (185/474), 21.6% (76/352), 29.1% (102/350) and 39.7% (92/232) of the patients tested, respectively. Of 156 patients with confirmed TB, 65.4% were LAM positive. Of those classified as non-TB, 84.0% were LAM negative. Adding LAM increased the diagnostic yield of the algorithms by 36.6%, from 47.4% (95%CI:39.4–55.6) to 84.0% (95%CI:77.3–89.4%), when using clinical signs and X-ray; by 19.9%, from 62.2% (95%CI:54.1–69.8) to 82.1% (95%CI:75.1–87.7), when using clinical signs and microscopy; and by 13.4%, from 74.4% (95%CI:66.8–81.0) to 87.8% (95%CI:81.6–92.5), when using clinical signs and Xpert. LAM positive patients had an increased risk of 2-months mortality (aOR:2.7; 95%CI:1.5–4.9).

**Conclusion:**

LAM should be included in TB diagnostic algorithms in parallel to microscopy or Xpert request for HIV-positive patients either ambulatory (severely ill or CD4<200cells/μl) or hospitalized. LAM allows same day treatment initiation in patients at higher risk of death and in those not able to produce sputum.

## Background

Diagnosis of tuberculosis (TB) remains a challenge in resource-limited countries and particularly in HIV-positive patients. Current diagnostic tools include sputum smear-microscopy and GeneXpert MTB/RIF (Xpert) assay [1]. However, despite the scale-up of Xpert implementation, the technical requirements of this assay restrict it to health facilities with laboratory services which are not always available in peripheral health facilities [2]. In addition, some patients with symptoms of TB are not able to produce sputum and these tests cannot be performed. Therefore, for many patients the diagnosis continues to be based on clinical signs and radiology (when available) despite their poor performance [3,4].

Alere Determine^™^ TB LAM Ag lateral flow strip (LAM) is an immunochromatographic test for the qualitative detection of lipoarabinomannan antigen of Mycobacteria. It is a point of care test performed on urine that provides results in 25 minutes. Urinary LAM detection represents haematogenously disseminated renal TB [5]. Therefore, LAM utility is restricted to HIV-positive patients with advanced immunodeficiency who are more likely to present TB dissemination. Using urine samples represent an advantage for the diagnosis of TB in HIV-positive patients in severe clinical condition not able to produce sputum [6]. The sensitivity of the assay is poor in HIV-negative patients and depends on the CD4 count in HIV-positive patients ranging from 39–67% in patients with CD4 below 200 cells/μl. The specificity is high, over 95% in majority of the studies [7]. In a meta-analysis of five studies, the pooled sensitivity and specificity were 45% and 92% respectively [8].

Although the accuracy of the LAM assay has been well evaluated, less is known about the value of incorporating LAM into a diagnostic algorithm in ambulatory and hospitalized HIV-positive patients. WHO has recently released a policy guidance on the use of LAM stating that the test may be used to assist in the diagnosis of TB in HIV-positive adults with symptoms of TB who have a CD4 cell count less than or equal to 100 cells/μL or who are seriously ill [9]. In the recently published algorithms for managing people living with HIV and suspected of having TB LAM is only mentioned as a possibility in seriously ill patients and the place of LAM within the algorithm is not clearly stated [10]. The primary objective of the study was to assess the incremental diagnostic yield of adding LAM to different diagnostic algorithms based on clinical signs, sputum smear-microscopy, chest X-ray and Xpert in HIV-positive patients with symptoms of pulmonary TB (PTB). We evaluated the incremental diagnostic yield in hospitalized and in ambulatory HIV positive patients as well as in subgroups of patients according to their immunological and clinical features. In addition, we explored the association between a positive LAM result and mortality at 2 months.

## Methods

### Design and population

This was a prospective cohort diagnostic study. Eligible patients were consecutive HIV-positive adults (≥15 years) who were hospitalized in the in-patients department or who attended the out-patients TB clinic, and who presented with cough for more than 2 weeks or with any cough and at least one of the following signs: loss of weight, night sweats, or fever (i.e. PTB suspicion). All hospitalized patients and the ambulatory patients who were either severely ill, or who had a CD4 count below 200cells/μl or a Body Mass Index (BMI) below 17Kg/m^2^ were included. Written informed consent was requested prior to inclusion. Exclusion criteria were intake of fluoroquinolones or anti-tuberculosis drugs in the month prior to the consultation. Being on Isoniazid Preventive Therapy was not an exclusion criterion.

### Sites

The study was conducted in Homa Bay County Hospital which is the referral health facility for a county of 800,000 people. Kenya is a high TB burden country with an incidence of 246/100,000 in 2014 and the Nyanza region where Homa Bay is located is the area with the highest case load reported in the country [11]. In 2013, overall HIV prevalence among people aged 15–49 years was estimated at 6.0% but it was 25.7% in Homa Bay, more than four times the national average in the country [12]. HIV prevalence reaches 74% among TB patients in this area [13]. TB and HIV care is supported by Médecins Sans Frontières and the Ministry of Health and is free of charge. HIV testing is proposed to all patients with symptoms of TB.

### Procedures

All patients were symptomatic and seeking care. On the first day (Day 1) of admission or consultation, patients had a clinical exam, a chest X-ray and a LAM test (performed by the study clinical officer during the patient’s consultation following the manufacturer recommended procedure). In addition, two sputum samples (spot and early morning) were collected through spontaneous expectoration or induced sputum. The spot sample was processed for smear microscopy, Xpert MTB/RIF assay and *Mycobacterium tuberculosis complex* (MTBC) culture. The early morning sample was processed for microscopy and MTBC culture. For patients not able to produce sputum, Xpert and MTBC culture were performed in centrifuged urine from the same sample initially collected for LAM. Smear microscopy and Xpert results were given the same day or the day after sample collection. Sputum and urine samples were centrifuged at 3000 rpm for 15–20 minutes and decontaminated using 2% NALC sodium hydroxide method. At least 0.5 ml of the sediment was resuspended in a conical tube by adding 1.5ml of Xpert MTB/RIF sample reagent. The suspension was incubated for 15 minutes at room temperature before being added to a cartridge and processed. The test was repeated up to two times using a new cartridge in case of invalid, error or no result. MTBC culture was performed in parallel using 2 methods: Thin Layer Agar (TLA) and Lowenstein-Jensen (LJ). TLA method consists of plates of 7H11agar-based medium read by conventional microscope [14]. Sputum specimens were decontaminated and the sediment was inoculated in one TLA plate and in one LJ slant. TLA was incubated at 37°C in a 5% CO2 incubator and LJ at 37°C in a standard incubator. Para-nitrobenzoic acid (PNB) was included in the TLA plates for simultaneous Non-tuberculous mycobacteria (NTM) detection while the antigen test MPT64 was used for the identification of *M*.*tuberculosis* complex growth in LJ. Broad spectrum antibiotics targeting community-acquired pneumonia were administered when TB was not initially diagnosed or in addition to TB treatment. Patients not started on TB treatment were reassessed clinically at a second consultation (Day 5). Outcomes of all patients whether on TB treatment or not, were assessed at 2 months (Month 2). Patients missing appointments and patients with positive MTBC culture result who had not been started on TB treatment were traced and asked to come to the clinic.

### Diagnostic algorithms

Diagnostic algorithms were constructed using the combination of different diagnostic tools among clinical examination, chest X-ray, urine LAM, sputum smear microscopy and XpertMTB/RIF assay in sputum and urine. The clinical officer made the decision to start TB treatment first based on clinical signs, then on X-ray, then on the LAM test result (once the test was performed) and finally using the smear microscopy and Xpert MTB/RIF assay results (once the results where available). Patients with clinical or radiological signs suggestive of TB as per clinician judgement or with positive LAM, smear microscopy or Xpert were started on TB treatment. Patients diagnosed by a given algorithm were considered those with a decision to start TB treatment.

### Definitions

Severely ill: patient presenting at least one of the following signs: temperature higher than 39°C, respiratory rate higher than 30 respirations/minute, cardiac rate higher than 120 beats minute, or unable to walk without help.

Very poor clinical condition: combination of being hospitalized, severely ill and having a BMI below 17Kg/m^2^.

LAM test result: Positive when a line grade 2 or above appeared; Negative when no band appeared or the line was grade 1 using the Reference Scale Card provided by the manufacturer (5-grades reference scale card).

Xpert MTB/RIF result: Positive when MTB was detected; Negative when MTB was not detected; Inconclusive when the test was not performed or the result reported as “invalid”, “error” or “no result”.

MTB culture result: Positive when at least one positive culture result was found in any of the collected sputum or urine specimens; Negative when all culture results from collected sputum specimens were negative; Inconclusive when culture results were contaminated or not performed (this includes cultures with partially missing results).

TB diagnosis (primary endpoint): Diagnosed of TB when a decision to start TB treatment was made by the clinician based on clinical signs or radiological features suggestive of TB or based on positive LAM, smear-microscopy or Xpert tests; Not diagnosed of TB when the decision was made to do not start TB treatment.

Confirmed TB case (reference standard): positive MTB culture or positive Xpert in any of the sputum or urine specimens collected; non-TB case: negative MTB culture and negative Xpert results from all sputum specimens collected; Inconclusive case: inconclusive MTB culture or Xpert result (including partially missing results).

### Statistical analyses

For primary analyses, the diagnostic yield was calculated as the proportion of confirmed TB patients diagnosed by a test or a diagnostic algorithm. The incremental diagnostic yield of adding LAM was the difference in the proportion of confirmed TB patients diagnosed by the algorithm with LAM compared to the algorithm without LAM. The incremental diagnostic yield was also assessed in the following study populations: i) hospitalized patients; ii) ambulatory patients iii) hospitalized patients in very poor condition; iv) ambulatory patients severely ill; v) hospitalized and ambulatory patients with CD4 below 200cells/μl; and vi) hospitalized and ambulatory patients severely ill. In addition, we report the proportions of positive LAM, Xpert and culture among all included patients and the reasons to start TB treatment among the patients started on TB treatment.

The diagnostic yields were calculated for independent populations and presented with exact binomial 95% confidence intervals (95% CI). However, the comparison of the diagnostic yield of algorithms with and without LAM was assessed in the same patients; a McNemar test for matched data was used. In addition, we estimated the sensitivity and specificity of the LAM test alone using the definitions of TB confirmed case and non-TB case as reference.

For the analysis on the association of the LAM results and the mortality at 2 months, multivariable logistic regression models included the following variables: age (per 1 year increase), sex (women vs men), clinical severity (being severely ill or not), BMI (<17Kg/m^2^ vs ≥17Kg/m^2^), CD4 (<200 cells/μl vs ≥200 cells/μl), ever initiated ART (yes vs no), LAM result (positive vs negative) and TB confirmation (positive, inconclusive vs negative). Variables with p<0.20 in univariate analyses were included in the multivariate ones. A decrease step-wise method was used, variables with p<0.10 were kept in the final model. Data were analyzed using Stata^®^ 13 software (College Station, Texas, USA).

### Ethical considerations

The study protocol was approved by the KEMRI/Scientific and Ethics Review Committee in Kenya and the Comité de Protection des Personnes (CPP), Saint Germain en Laye, France.

## Results

### Description of the population and patient flow

**Fig 1:**
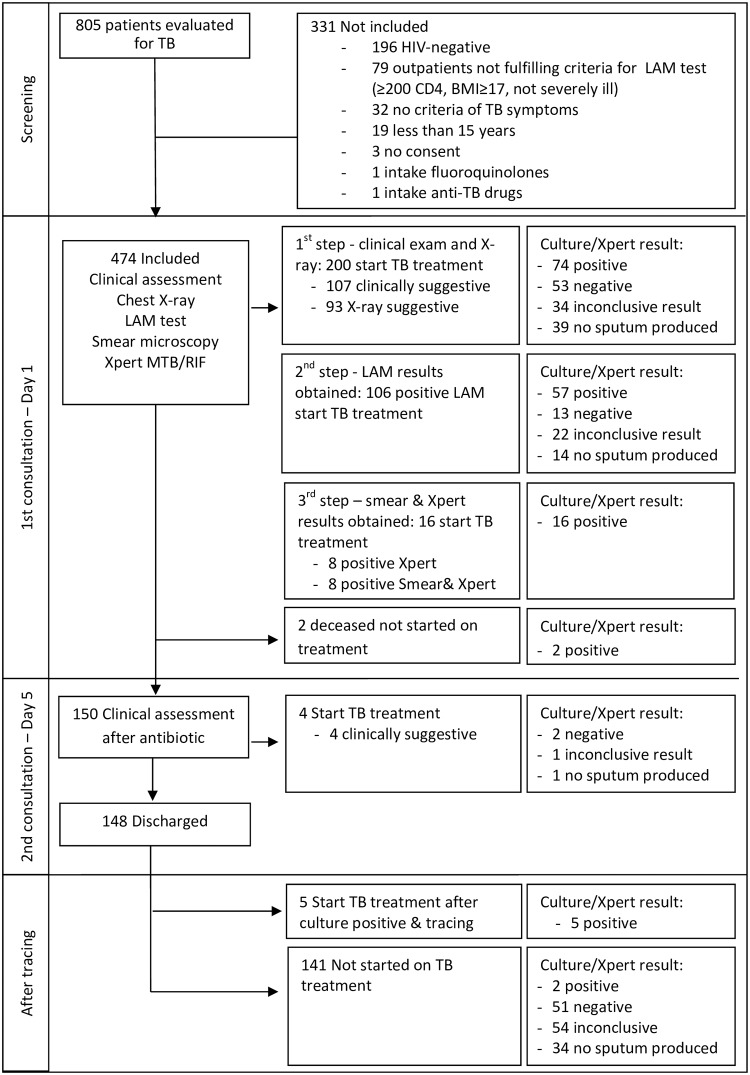
Diagnostic and treatment pathway of 474 patients with TB symptoms.

Between October 2013 and August 2015, 474/805 (58.9%) patients were included in the study and of them 331/474 (69.8%) patients were started on TB treatment (Fig 1). Median age was 35 years (IQR: 29–44) and 244 (51.5%) were women. In total, 360 (76.0%) patients were hospitalized and 174 (24.0%) were ambulatory patients (Table 1). Median CD4 count was 109 cells/μl (IQR: 43–214), 282 (59.5%) patients had ever initiated ART and of them 268 were on ART. The distribution of the patients according to the level of CD4 count was: 132 (28.6%) ≥200 cells/μl, 132 (28.6%) 100–199 cells/μl, 70 (15.2%) 50–99 cells/μl, 128 (27.7%) <50 cells/μl. While all patients were able to produce urine, 122/474 (25.7%) were not able to produce sputum spontaneously and 110/474 (23.2%) patients could not produce sputum after sputum induction. A total of 156 (56.7%) patients were confirmed with TB. The reasons to start TB treatment among the 331 started on TB treatment were: 111 (33.5%) suggestive clinical signs, 106 (32.0%) positive LAM result, 93 (28.1%) suggestive chest X-ray, 16 (4.8%) positive Xpert result and 5 (1.5%) positive culture result.

**Table 1:** Patients’ characteristics of the 474 HIV-positive patients with TB symptoms included.

	*All patients*	*In-patients*	*Out-patients*^1^
	*(N = 474)*	*(N = 360)*	*(N = 114)*
Age, median years (IQR)	35 (29–44)	35 (28–45)	35 (30–43)
Women, n (%)	244 (51.5)	185 (51.4)	59 (51.8)
History of TB, n (%)	117 (24.7)	86 (23.9)	31 (27.4)
Antibiotic in the past 2 weeks, n (%)	103 (21.7)	80 (22.2)	23 (20.2)
BMI^2^, median (IQR)	17 (16–20)	18 (16–20)	17 (16–18)
BMI<17Kg/m^2^, n (%)	209 (44.1)	138 (38.3)	71 (62.3)
Severely ill, n (%)	402 (84.8)	334 (92.8)	68 (59.7)
CD4 <200cells/μl, n (%)	330 (69.6)	242 (67.2)	88 (77.2)
ART ever initiated^3^, n (%)	282 (59.9)	218 (60.9)	64 (56.6)
Not able to produce sputum spontaneously	122 (25.7)	111 (30.8)	11 (9.6)
LAM positive, n (%)	185 (39.0)	151 (41.9)	34 (29.8)
Smear positive^4^, n (%)	76 (21.6)	48 (19.2)	28 (27.5)
Xpert positive^4^, n (%)	115 (31.5)	72 (27.6)	43 (41.4)
Culture positive^4^, n (%)	107 (43.3)	71 (43.3)	36 (43.4)
Xpert or culture positive^4^, n (%)	156 (56.7)	106 (57.9)	50 (54.4)

^1.^ Out-patients inclusion criteria were: CD4<200 or severely ill or BMI<17

^2.^ BMI: Body Mass Index

^3.^ ART: Antiretroviral therapy

^4.^ Results available: Smear in 250 and 102 In- and Out-patients; Xpert in 261 and 104 In- and Out-patients; Culture in 164 and 83 In- and Out-patients; Xpert or culture in 183 and 92 In- and Out-patients. Xpert and culture results include sputum and urine samples.

### Laboratory results and accuracy of LF-LAM assay

Considering the 474 patients included, 185 (39.0%) had a positive LAM result, 102 (21.5%) a positive Xpert result in sputum, 92 (19.4%) a positive MTBC culture result in sputum and 76 (16.0%) a positive smear microscopy result. Smear microscopy, Xpert and MTBC culture in sputum were positive in 21.6% (76/352), 29.1% (102/350) and 39.7% (92/232) of the patients tested, respectively (Table 2). There wasn’t any case with non-tuberculosis mycobacteria detected. In urine, Xpert and culture were positive in an additional 13.5% (13/96) and 14.2% (15/104) of the patients tested, respectively. Including the positive urine results, Xpert was positive in 31.5% (115/365) patients, culture in 43.3% (107/247) and Xpert or culture in 56.7% (156/275).

**Table 2:** LAM, smear microscopy, Xpert and MTB culture results.

	LAM		
	Positive	Negative	Total
	(N = 185)	(N = 289)	(N = 474)
	n (%)	n (%)	n (%)
Smear microscopy			
Positive	57 (75.0)	19 (25.0)	76 (100)
Negative	71 (25.7)	205 (74.3)	276 (100)
Missing	57 (46.7)	65 (53.3)	122 (100)
Xpert^1^			
Positive	81 (70.4)	34 (29.6)	115 (100)
Negative	58 (23.2)	192 (76.8)	249 (100)
Missing^2^	46 (42.2)	63 (57.8)	110 (100)
Culture^1^			
Positive	59 (55.1)	48 (44.9)	107 (100)
Negative	33 (23.6)	107 (76.4)	140 (100)
Missing^2^	93 (41.0)	134 (59.0)	227 (100)
Xpert & Culture^1^			
Positive	102 (65.4)	54 (34.6)	156 (100)
Negative	19 (16.0)	100 (84.0)	119 (100)
Missing^2^	64 (32.2)	135 (67.8)	199 (100)

^1.^ Includes sputum and urine results

^2.^ At least one sample with no result or contaminated result (culture) in the absence of a positive result

The sensitivity and specificity of LAM was 65.4% (95%CI: 57.4–72.8) and 84.0% (95%CI: 76.2–90.1) respectively. The accuracy of LAM varied in hospitalized and ambulatory patients. The sensitivity and specificity was 68.5% (95%CI: 59.1–77.5) and 77.9% (95%CI: 67.0–86.6) respectively in hospitalized patients and 58.0% (95%CI: 43.2–71.8) and 95.2% (95%CI: 83.8–99.4) respectively in ambulatory patients. The 19 patients with a LAM positive and Xpert and culture negative results were all in severe clinical condition. Chest X-ray was reported as suggestive of TB for 8 patients, as possible TB for 8 patients and as no TB for 1 patient. LAM results were reported as grade 2 for 7 patients, grade 3 for 6 patients and grade 4 for 6 patients.

Among patients with no sputum sample, 50/110 (45.5%, 95%CI: 36.3–55.0) had a positive LAM test compared to 135/364 (37.1%, 95%CI: 32.3–42.2) among those with a sputum sample. Of the 82 patients not able to produce sputum for whom LAM and Xpert results in urine were available, 39 (47.6%) had a positive LAM result and 12 (14.6%) a positive Xpert result in urine (p = 0.039). Among the 21 patients Xpert or culture positive in urine, 16 (76.2%) had a positive LAM result. Among the 59 patients Xpert and culture negative in urine, 23 (39.0%) had a positive LAM result.

### Incremental yield of adding LAM to the diagnostic algorithms

Adding LAM test increased by 36.6% and 30.8% the proportion of confirmed TB cases diagnosed that would have been otherwise missed by an algorithm based on clinical signs and chest X-ray or on smear microscopy alone, p<0.001 (Table 3). LAM combined with microscopy had a higher diagnostic yield than each of the tools separately and led to a diagnosis in 76.3% (95%CI: 68.8–82.7) of the confirmed TB patients. Adding LAM increased by 19.9% the diagnostic yield of an algorithm based on clinical signs and sputum smear-microscopy from 62.2% (95%CI: 54.1–69.8) to 82.1% (95%CI: 75.1–87.7), p<0.001. The diagnostic yield of an algorithm including clinical signs, smear microscopy and LAM was similar to the yield of an algorithm including clinical signs, Xpert in sputum and X-ray (83.3%, 95%CI: 76.5–88.8). Adding LAM increased by 13.4% the diagnostic yield of an algorithm including clinical signs and Xpert in sputum with the proportion of confirmed TB patients started on appropriate TB treatment raising from 74.4% (95%CI: 66.8–81.0) to 87.8% (95%CI: 81.6–92.5). Clinical exam, X-ray, Xpert in sputum and LAM diagnosed almost all patients with confirmed TB (94.2%, 95%CI: 89.3–97.3).

**Table 3:** Diagnostic yield of different diagnostic algorithms with no LAM and with LAM in 156 TB confirmed patients.

		Diagnostic yield of the algorithm with no LAM	Diagnostic yield of the algorithm with LAM	Incremental yield
		% (95%CI)^1^	% (95%CI)	%
No Xpert	-	-	65.4 (57.4–72.8)	-
	Clinical signs & Xray	47.4 (39.4–55.6)	84.0 (77.3–89.4)	36.6
	Smear microscopy	45.5 (37.5–53.7)	76.3 (68.8–82.7)	30.8
	Clinical signs & smear microscopy	62.2 (54.1–69.8)	82.1 (75.1–87.7)	19.9
	Clinical signs & smear microscopy & Xray	73.1 (65.4–79.9)	89.1 (83.1–93.5)	16.0
Xpert	Xpert in sputum	64.7 (56.7–72.2)	85.3 (78.7–90.4)	20.6
	Xpert in sputum-urine^2^	73.7 (66.1–80.4)	85.3 (78.7–90.4)	11.6
	Clinical signs & Xpert in sputum	74.4 (66.8–81.0)	87.8 (81.6–92.5)	13.4
	Clinical signs & Xpert in sputum-urine^2^	81.4 (74.4–87.2)	89.1 (83.1–93.5)	7.7
	Clinical signs & Xpert in sputum & Xray	83.3 (76.5–88.8)	94.2 (89.3–97.3	10.9
	Clinical signs & Xpert in sputum-urine^1^ & Xray	89.1 (83.1–93.5)	95.5 (91.0–98.2)	6.4

^1.^ Exact binomial 95% confidence intervals (95%CI)

^2.^ Xpert in sputum for patients with sputum sample and in urine for those not able to produce sputum

Regarding TB treatment of patients classified as non-TB, 46.2% (95%CI: 38.7–57.2) of the non-TB patients were started on treatment using clinical exam and X-ray, 33.6% (95%CI: 25.2–42.8) using clinical exam, smear microscopy and LAM, 21.0% (95%CI: 14.1–33.2) using clinical exam and Xpert in sputum, 16.8% (95%CI: 10.6–24.8) using smear microscopy and LAM, and 16.0% (95%CI: 9.9–23.8) using Xpert in sputum and LAM.

### Incremental diagnostic yield of LAM in study subgroups

The incremental diagnostic yield of adding LAM to an algorithm varied according to the population (Table 4). In hospitalized patients, LAM increased the diagnostic yield of an algorithm based on clinical signs and smear-microscopy by 23.6% (p<0.001) and of an algorithm based on clinical signs and Xpert in sputum by 17.0% (p<0.001). However, in ambulatory patients, the incremental yield of adding LAM to the algorithm was statistically significant only for the algorithms with no Xpert (12.0% increase when LAM was added to an algorithm based on clinical signs and smear-microscopy, p = 0.03).

**Table 4:** Diagnostic yield of different diagnostic algorithms with no LAM and with LAM among TB confirmed patients from various study subgroups.

	Overall	Hospitalized	Outpatients^1^	Hospitalized in very poor condition^2^	Outpatients severely ill	Patients with CD4<200	Patients severely ill^3^
	(N = 156)	(N = 106)	(N = 50)	(N = 39)	(N = 37)	(N = 129)	(N = 139)
	%	%	%	%	%	%	%
	(95%CI)^4^	(95%CI)	(95%CI)	(95%CI)	(95%CI)	(95%CI)	(95%CI)
LAM alone	65.4	68.9	58.0	76.9	59.5	68.2	66.9
	(57.4–72.8)	(59.1–77.5)	(43.2–71.8)	(60.7–88.9)	(42.1–75.2)	(59.4–76.1)	(58.4–74.6)
Clinical signs & LAM	76.9	73.6	84.0	82.1	91.9	80.6	79.1
	(69.5–83.3)	(64.1–81.7)	(70.9–92.8)	(66.5–92.5)	(78.1–98.3)	(72.7–87.0)	(71.4–85.6)
Clinical signs & smear microscopy	62.2	54.7	78.0	53.8	81.1	63.6	62.6
	(54.1–69.8)	(44.8–64.4)	(64.0–88.5)	(37.2–89.7)	(64.8–92.0)	(54.6–71.9)	(54.0–70.6)
Clinical signs & smear microscopy & LAM	82.1	78.3	90.0	82.1	91.9	82.9	82.7
	(75.1–87.7)	(69.2–85.7)	(78.2–96.7)	(66.5–92.5)	(78.1–98.3)	(75.3–89.0)	(75.4–88.6)
Incremental yield	19.1	23.6	12.0	28.3	10.8	19.3	20.1
Clinical signs & smear microscopy & X-ray	73.1	67.9	84.0	64.1	89.2	72.9	73.4
	(65.4–79.9)	(58.2–76.7)	(70.9–92.8)	(47.2–78.8)	(74.6–97.0)	(64.3–80.3)	(65.2–80.5)
Clinical signs & smear microscopy & X-ray & LAM	89.1	86.8	94.0	84.6	97.3	89.1	89.2
	(83.1–93.5)	(78.8–92.6)	(83.5–98.7)	(69.5–94.1)	(85.8–99.9)	(82.5–93.9)	(82.8–93.8)
Incremental yield	16.0	18.9	10.0	20.5	8.1	16.2	15.8
Clinical signs & Xpert in sputum	74.4	66.0	92.0	64.1	89.2	75.2	73.4
	(66.8–81.0)	(56.2–75.0)	(80.8–97.8)	(47.2–78.8)	(74.6–97.0)	(66.8–82.4)	(65.2–80.5)
Clinical signs & Xpert in sputum & LAM	87.8	83.0	98.0	84.6	97.3	87.6	87.8
	(81.6–92.5)	(74.5–89.6)	(89.4–99.9)	(69.5–94.1)	(85.8–99.9)	(80.6–92.7)	(81.1–92.7)
Incremental yield	13.4	17.0	6.0	20.5	8.1	12.4	14.4
Clinical signs & Xpert in sputum & X-ray	83.3	77.4	96.0	71.8	94.6	82.9	82.0
	(76.5–88.8)	(68.2–84.9)	(86.3–99.5)	(55.1–85.0)	(81.8–99.3)	(75.3–89.0)	(74.6–88.0)
Clinical signs & Xpert in sputum & X-ray & LAM	94.2	91.5	100	87.2	100	93.0	93.5
	(89.3–97.3)	(84.5–96.0)	(92.9–100)	(72.6–95.7)	(90.5–100)	(87.2–96.8)	(88.1–97.0)
Incremental yield	10.9	14.1	4.0	15.4	5.4	10.1	11.5

^1.^ Outpatients inclusion criteria were: CD4<200, severely ill or BMI<17

^2.^ Very poor condition: CD4<200 and severely ill and BMI<17

^3.^ Severely ill: temperature >39°C, respiratory rate >30 respirations/min, cardiac rate >120 beats/min, or unable to walk without help.

^4.^ Exact binomial 95% confidence intervals (95%CI)

Regarding the immunological and clinical features, the incremental diagnostic yield of adding LAM was greater in patients hospitalized in very poor clinical condition (severely ill and BMI<17kg/m^2^ and CD4 count<200cell/μl). In addition, there were no differences in the incremental yield of adding LAM in all ambulatory patients (using both CD4 and clinical selecting criteria) compared to ambulatory patients severely ill (using only clinical selecting criteria).

### Predictors of mortality at 2 months

Of the 474 enrolled participants, vital status at 2 months was ascertained in 468 (99%) and 65/468 (13.9%) patients had died. Median time to death was 14 (IQR: 5–24) days. Mortality was higher in patients with confirmed TB compared to non-TB patients: 17.4% vs 5.9% (p = 0.004) and in patients LAM positive compared to those LAM negative: 22.8% vs 8.1% (p<0.0001). Although not statistically significant, there was a trend of higher mortality in LAM positive patients (vs LAM negative) among confirmed TB patients: (22.8% vs 11.1%, p = 0.130). A LAM-positive result was associated with higher mortality in non-TB patients (15.8% in LAM positive vs 4.0% in LAM-negative, p = 0.047) and in patients with inconclusive classification of TB (28.1% in LAM-positive vs 9.9% in LAM-negative, p = 0.001). In multivariate logistic regression analyses, patients with a urine LAM positive result had a 2.7 folds increased risk of mortality at 2 months compared with patients with negative LAM results patients (Table 5). However, there was not a dose response relationship between grade and mortality (aOR grade 2 = 2.0 [95%CI: 0.8–5.5]; aOR grade 3 = 3.3 [95%CI: 1.7–6.4]; aOR grade 4 = 2.3 [95%CI:1.0–5.4]).

**Table 5:** Factors associated with 2-months mortality in HIV-positive patients with symptoms of TB.

	*Univariate*			*Multivariate*		
				*(N = 468)*		
	OR	95% CI	p	aOR	95% CI	p
Age (/year)	1.0	0.9–1.0	0.882	-	-	-
Sex						
Men	Ref					
Women	0.6	0.4–1.1	0.092	-	-	-
ART ever initiated^1^						
No	Ref					
Yes	1.4	0.8–2.3	0.260	-	-	-
BMI^2^						
≥17Kg/m^2^	Ref					
<17Kg/m^2^	1.0	0.6–1.7	0.946	-	-	-
Severely ill						
No	Ref			Ref		
Yes	13.5	1.8–98.6	0.011	9.3	1.3–69.4	0.029
CD4 count						
≥200 cells/μl	Ref					
<200 cells/μl	1.9	1.0–3.6	0.049	-	-	-
TB treatment started						
No	Ref					
Yes	1.8	1.0–3.5	0.063	-	-	-
LAM result						
Negative	Ref			Ref		
Positive	3.4	1.9–5.8	<0.001	2.7	1.5–4.9	0.001
Culture/Xpert result						
Negative	Ref			Ref		
Positive	3.3	1.4–8.0	0.006	1.8	0.7–4.5	0.209
Inconclusive	3.0	1.3–7.0	0.012	2.2	0.9–5.3	0.076

^1.^ ART: Antiretroviral therapy

^2.^ BMI: Body Mass Index

## Discussion

In this study, LAM test increased by 36.6% the diagnostic yield of an algorithm based on clinical signs and X-ray, by 19.9% the yield of an algorithm based on clinical signs and sputum smear microscopy, and by 13.4% the yield of an algorithm based on clinical signs and Xpert. The use of LAM test alone allowed diagnosing 65% of the patients with confirmed TB. Furthermore, an algorithm based on clinical signs, sputum smear-microscopy and LAM led to an appropriate TB diagnosis of 82% of the patients with confirmed TB, a similar proportion to an algorithm including clinical signs, Xpert in sputum and X-ray. Among hospitalized patients, the incremental yield of adding LAM was significant in algorithms with no Xpert (23.6% additional patients diagnosed using clinical signs and microscopy) and in algorithms with Xpert (17.0% additional patients diagnosed using clinical signs and Xpert). Among ambulatory patients, LAM alone diagnosed 58% of the patients with confirmed TB. Adding LAM led to 23.6% additional confirmed TB patients diagnosed using clinical signs and microscopy. One quarter of the patients could not produce sputum and few patients could produce sputum after sputum induction probably due to their severe clinical condition. For them, TB diagnosis could only be done based on clinical signs, X-ray or LAM. In addition, positive LAM was a marker of increased risk of mortality.

Using LAM test alone as a rule-in screening test at first contact with the patient could diagnose an important proportion of confirmed TB cases. LAM test sensitivity was comparable with other settings [15–18]. In hospitalized HIV-positive patients with symptoms of TB, performing a LAM test on arrival to all of them would be relatively easy to implement as no selection of patients would be necessary. LAM result and clinical judgement would allow immediate adequate TB treatment in almost three quarters of the TB confirmed patients. The added value would be even greater considering that one third of the hospitalized patients could not produce sputum and the prognostic value of positive LAM to detect patients at higher risk of death. Among ambulatory patients, those severely ill and those with CD4 count below 200 cells/μl would benefit from LAM. Using only the clinical criteria of severity to select patients when CD4 is not available would be an appropriate strategy (similar diagnostic yield of LAM); however, it would exclude a proportion of patients (one quarter in our study) who could benefit from the test.

In places with access to smear microscopy and no Xpert on site, combining smear microcopy and LAM would lead to a higher diagnostic yield compared to smear microscopy alone [16,17,19,20]. An algorithm based on clinical signs, LAM and smear microscopy for HIV-positive patients with symptoms of TB could offer the possibility of a quick and affordable diagnosis of TB. In contexts with no smear microscopy, the algorithm could be based on clinical signs and LAM.

In places with access to Xpert but without immediate results (samples transferred elsewhere or results not obtained in the same day) LAM test could be done at the initial consultation while Xpert test is requested in parallel. Clinical signs and LAM would allow immediate treatment of an important part of the patients with confirmed TB while waiting for the Xpert test results. In places with access to Xpert and same day results, LAM test could have a role for hospitalized patients [17,21].

In all contexts, LAM could help to diagnose patients not able to produce sputum (26% in our study). In addition, LAM may be a marker of greater disease dissemination and severity. In line with other studies [16,18,22–25], in Kenya, LAM positive patients had a 3-folds higher risk of mortality at 2 months compared to those LAM negative. A recent systematic review and meta-analyses has shown a robust association between detectable LAM in urine and increased risk of mortality after adjusting for other risk factors for mortality [26].

Among patients not able to produce sputum, a higher proportion had a positive LAM test compared to Xpert in urine. However, LAM positive patients not able to produce sputum and with negative Xpert result in urine were excluded from the analyses on the diagnostic yield. If those LAM positive patients are true TB cases [20,27], the diagnostic yield of an algorithm including clinical signs, Xpert in sputum and LAM would be higher.

The proportion of non-TB patients started on treatment in an algorithm was consonant with the less specific diagnostic tools included in the algorithm. Algorithms including X-ray led the highest numbers of non-TB patients started on treatment while algorithms including only laboratory tests led to the lowest numbers.

A first limitation of this study was the use of a reference based only on sputum and urine samples. In this programmatic context were bronchoscopy and mycobacterial blood culture were not available, the specificity of LAM, which was lower among hospitalized patients than the values over 95% reported in other studies [15–17,28], may have been underestimated [29]. In addition, hospitalized patients are more likely to present disseminated TB which is difficult to confirm using sputum based diagnostic tests [30]. In line with this hypothesis, LAM positive patients had a higher mortality compared to LAM negative among individuals classified as non-TB or inconclusive diagnosis of TB. Cross reactions with NTM are not likely as the proportion of NTM in this setting seems to be very low. Based on the high specificity of LAM found in other studies, we think that the majority of the LAM positive patients may be true positive and that adding LAM to the algorithm would not represent an important cost in terms of additional patients unnecessarily treated. A second limitation is the fact that the algorithms were compared in the same population. Related to this, LAM test was read by the same clinician who had performed the clinical exam and who could therefore be biased by the clinical findings. Finally, patients who could not produce sputum were excluded from the analyses on diagnostic yield (unless they had a positive Xpert or culture result in urine). The real added value of LAM may have been underestimated since a proportion of these patients would have been diagnosed through LAM.

In conclusion, the incremental diagnostic yield of adding LAM to the algorithms suggests that LAM should be included in the algorithms for ambulatory (either severely ill or with CD4<200cells/μl) and hospitalized HIV-positive patients with symptoms of TB. Point of care urine LAM test alone would diagnose a considerable proportion of the patients with confirmed TB. In contexts with only smear microscopy available, an algorithm including clinical signs, smear microscopy and LAM would have a detection yield close to an algorithm including Xpert. In contexts with no immediate Xpert results, the urine LAM test would allow same day diagnosis and treatment of a high proportion of TB confirmed patients while waiting for the Xpert results. In settings with Xpert available on site and immediate results, LAM would be useful in hospitalized patients and could be done in parallel to the Xpert request. In all contexts, the use of urine LAM test would help TB diagnosis in a considerable proportion of patients who are not able to produce sputum and for whom the diagnosis can only be based on clinical signs and on chest X-ray (when available). In addition, LAM could lead to quick treatment initiation in patients at higher risk of death.

## Supporting Information

S1 File Dataset. (DTA)
